# Quality evaluation questionnaires – nursing homes (QEQ-NH); validation of questionnaires for measuring quality of care in nursing homes from various perspectives

**DOI:** 10.1186/s12913-021-06823-4

**Published:** 2021-09-13

**Authors:** Mattanja Triemstra, Juliane Menting, Bellis van den Berg

**Affiliations:** 1 Nivel, Netherlands Institute for Health Services Research, PO Box 1568, 3500 BN Utrecht, The Netherlands; 2 Vilans, Netherlands Centre of Expertise for Long-term Care, Utrecht, The Netherlands

**Keywords:** Quality of care, Nursing homes, Questionnaires, Validity, Cognitive interviews

## Abstract

**Background:**

This study aims to describe the validation and optimization of a new instrument designed to comprehensively measure and evaluate the quality of care in nursing homes; the *Quality Evaluation Questionnaires for Nursing Homes* (QEQ-NH). This instrument comprises several questionnaires on the perceived quality of care for various perspectives (e.g. clients, family and professional caregivers) and covers eight themes of the national quality framework for nursing home care in the Netherlands.

**Methods:**

Data were collected in six nursing homes between September 2017 and June 2018, among 359 residents, 48 family caregivers and 648 professional caregivers who completed a subgroup-specific questionnaire of the QEQ-NH. Construct and criterion validity of the three questionnaires were tested with item- and scale analyses. Content validity of the questionnaires was tested in cognitive interviews with 20 participants (7 residents, 5 family caregivers and 8 professional caregivers).

**Results:**

Psychometric analyses confirmed the multidimensionality and reliability of the three questionnaires, and the cognitive interviews showed various possibilities for further optimization of the instrument. Construct, criterion and content validity of the three questionnaires ranged from acceptable to good. Cronbach’s alphas were > .70 for almost all scales. More than half of the items were candidate for optimization according to the cognitive interviews, mainly due to clarity or knowledge problems, and the questionnaires of the QEQ-NH were optimized accordingly.

**Conclusions:**

The Quality Evaluation Questionnaires for Nursing Homes (QEQ-NH) provide a solid basis to measure the quality of nursing home care, by covering the national quality themes and by integrating the various perspectives of all parties involved. With real-time feedback, the instrument provides the management and care teams with information to select possibilities or areas for improvement and to continuously monitor the effects of quality improvement in nursing homes.

**Supplementary Information:**

The online version contains supplementary material available at 10.1186/s12913-021-06823-4.

## Background

### Quality of nursing home care

Quality assurance and improvement is a major and continuous challenge in nursing homes. These long-term care facilities aim to provide person-centred, safe and effective care to their residents, in order to maximize their quality of life. Good care relationships and personalized care are likely to yield positive experiences and high quality of care in long-term care settings [1–3]. However, the quality of care remains under pressure due to an increasing elderly population, limited resources and labour shortages that negatively affect staff capacity and responsive workforces [4]. Necessary reforms, budget cuts and a restricted access to nursing homes may also be at the expense of the perceived quality. For example in the Netherlands, the availability of staff was found to be a major concern of residents and family caregivers [5] and the care is often perceived as hurried [6]. Given these ongoing strains, the question on how to maintain or even improve the quality of care in nursing homes remains topical. In response, continuous monitoring, learning and improvement are needed.

#### Quality standards and framework

Quality standards and frameworks aim to serve as guides for providing good quality of care in nursing homes, in order to guarantee the quality and safety of services and to optimize the quality of life of residents. In 2016, Ireland has introduced national standards for residential care settings for older people [7], which served as an example for the national quality framework on nursing home care for the Netherlands [8]. These standards are based on literature and the experience and knowledge of various experts and stakeholders. See Table 1 for the eight themes of the Dutch quality framework, reflecting the *content* of care (themes 1–4) and *preconditions* for quality (themes 5–8). Learning and improving quality (theme 4) is the central theme of the quality framework. Nursing homes are responsible for the measurement of client experiences at least once a year, in order to transparently report the results and to continuously improve the personalized care and well-being of their residents [8].

**Table 1:** Themes of the national quality framework for nursing home care in the Netherlands (2017)*

Theme:	Description:
**1 Person-centered care**	How the nursing home places residents in the centre of what they do.
**2 Living and well-being**	How nursing homes identify, support and promote the quality of live and well-being for residents and their informal caregivers.
**3 Safety**	How nursing homes guarantee basic safety for residents by following professional standards and guidelines, and through preventing and minimizing harm and learning from safety incidents.
**4 Learning and improving quality**	How nursing homes dynamically learn and improve to provide optimal care for residents, using best available evidence and information.
**5 Leadership, governance and management**	The arrangements put in place by the nursing home for accountability, decision-making, risk management and meeting its strategic, legal and financial obligations.
**6 Responsive workforce**	Adequate and sufficient staff with the necessary numbers, skills and abilities to respond to the needs of residents.
**7 Use of resources**	Using resources effectively and efficiently to deliver best achievable outcomes for the money and resources used.
**8 Use of information**	Actively using information as a resource for planning, delivering, monitoring, managing and improving services, and transparency of quality information for clients, family and society.

*Source: Zorginstituut Nederland, 2017 [8]

#### Measuring the quality of care in nursing homes

Valid instruments are needed to assess relevant quality indicators and to capture the perceived quality of care from different perspectives, in order to continuously monitor and improve the quality of nursing home care. Besides the commonly registered safety and clinical outcome indicators (e.g. medication errors, pressure ulcer scores, urinary tract infections, injurious falls), other instruments may additionally provide insight into the experiences of all stakeholders involved in the caring relationship (i.e. the residents, their family and the professional caregivers) in order to realize responsive care that fits the various needs.

Many quantitative instruments have been developed to register and monitor the quality of care, quality of life and client experiences in nursing homes. Since 2007, seven reviews have highlighted altogether at least 75 internationally used instruments for measuring quality of life or satisfaction of nursing home residents [9–15]. Most of these instruments focus on specific domains of functioning (e.g. psychological well-being or cognitive functioning) or on specific conditions such as dementia. Only few instruments measure the perceived quality of nursing homes or residential care, from the perspective of either residents themselves or their family caregivers. A recent example is the Quality of Care Experience (QCE) questionnaire, which has been validated in Australia as a new measure of quality of care experienced in aged care [16]. This short questionnaire measures the quality of care experience from the perspective of older people and family carers in residential aged care and home care settings. But specifically for nursing homes, there seems to be a lack of instruments covering the various aspects of quality of care, as well as combining the perspectives of all relevant stakeholders (e.g. residents, family and care staff). As far as we know, no instrument has previously been developed and validated that could provide such an integrated picture of the quality of care in nursing homes from various perspectives.

Like many other countries, the Netherlands has a long tradition of measuring the quality of care in nursing home care in a quantitative way. For example, with risk assessments for the national Prevalence Measurement of Care Problems in Care Homes [17], or the measurement of client experiences with the Consumer Quality Index (CQ-index) [5] which is partly based on the American CAHPS® Nursing Home Survey [18]. Over the last decade, there has been a shift towards more qualitative, narrative or mixed methods aiming to fully capture the construct of experienced quality of care. Furthermore, the growing awareness that quality is not just static but continuously needs discourse, maintenance and involvement of all stakeholders, has led to the development of more dynamic and comprehensive instruments. One of these new and frequently used instruments is the ‘Quality Evaluation Questionnaires – Nursing Homes’ (QEQ-NH).

### Aim of the study

This study aims to describe, validate and optimize the Quality Evaluation Questionnaires for Nursing Homes (QEQ-NH). This comprehensive instrument has not been published nor validated before, but is already widely used in the Netherlands. The study focuses on the validation and optimization of three QEQ-NH questionnaires that measure quality of nursing home care from three main perspectives (i.e. residents, family and professional caregivers), by answering the following two research questions:

1. *What is the construct, criterion and content validity of the QEQ-NH questionnaires?*
2. *How could the questionnaires be further optimized?*

## Method

Three Quality Evaluation Questionnaires for Nursing Homes (QEQ-NH) were extensively validated and optimized in a multistage approach, including both quantitative and qualitative testing, with psychometric testing and cognitive interviewing respectively. Although the optimal order for questionnaire development would have been to first conduct cognitive interviews as a pre-test, followed by a quantitative field test [19], the questionnaires under study were already in use. Consequently, we used data already collected in six nursing homes to conduct psychometric analyses and to select problematic items for cognitive testing.

### Quality evaluation questionnaires for nursing homes (QEQ-NH)

The QEQ-NH consists of several questionnaires for measuring the quality of care in nursing homes from different perspectives, including residents, family and professional caregivers as the main categories of respondents. The instrument has been developed in 2015–2017 by Vilans (the national Centre of Expertise for Long-term Care in the Netherlands), and is used annually in Dutch nursing homes.

The instrument has at least three distinctive features. First, it fully covers the Dutch national quality framework (see Table 1). By completing the questionnaire, respondents become more aware of the meaning of the framework in daily practice. Second, the instrument provides 360 degrees feedback by integrating the various perspectives of different parties involved (e.g. care professionals, residents, family, volunteers and managers). Third, it provides teams of caregivers and managers with meaningful and real-time feedback information via an online dashboard, for starting a dialogue within teams or with residents and family. This dialogue is the starting point for quality improvement in a short-cyclical way, in line with the PDCA-cycle (Plan, Do, Check, Act) for quality control [20]. The instrument enables care teams and the management to select opportunities or areas for quality improvement in nursing homes. Subsequently, the quality improvement efforts can be monitored and evaluated longitudinally.

The themes and number of items addressed in each questionnaire depend on the applicability and the ability of each subgroup to reflect on a certain topic of the Dutch quality framework for nursing homes. Questions are formulated as propositions and answering scales range from 1 (completely disagree) to 5 (completely agree). See Table 2 for an overview of domains and number of items, and Supplementary file 1 for a more detailed description of the content of the three questionnaires. The questionnaires are not under license and can be requested by contacting Vilans (third author).

**Table 2:** Domains and items of the Quality Evaluation Questionnaires - Nursing Homes (QEQ-NH)^#^

	Number of items per domain, per subgroup:		
Domains:	Residents	Family caregivers	Professional caregivers
**1 Person-centered care**	8	8	9
**2 Living and well-being**	7	8	8
**3 Safety**	3	3	12
**4 Learning and improving quality**	0	0	5
**5 Leadership, governance and management**	1	1	5
**6 Responsive workforce**	3	3	8
**7 Use of resources**	0	0	5
**8 Use of information**	1	1	2

# Original versions of the three questionnaires for residents, family caregivers or professional caregivers (Vilans, 2017)

The Net Promoter Score (NPS) is also part of the QEQ-NH questionnaires. The NPS reflects the likelihood of residents, family and professional caregivers to recommend the nursing home to others (family, friends or colleagues), and is used as a global indicator for the quality of care in nursing homes in the Netherlands [8]. The NPS-question was formulated as ‘*Would you recommend our nursing home to family, friends or colleagues?’,* with an answering scale from 0 to 10. Scores are categorized into *promoters* (9 or 10), *passives* (7 or 8), and *detractors* (0 to 6) [21]. To calculate the NPS, the percentage of detractors is subtracted from the percentage of respondents who are promoters: NPS = % Promoters (scores 9 or 10) - % Detractors (scores 0 to 6), resulting in a score between − 100 and +  100.

### Data collection and study population

Data were collected with the QEQ-NH questionnaires between September 2017 and June 2018, at the start of a quality improvement program in six nursing homes across the Netherlands. All nursing homes provide care for vulnerable elderly, of which a substantial part suffers from dementia. The six participating nursing homes were all middle sized, with about 100 to 300 residents, and had one to four residential locations. The questionnaires were distributed during a three-week period to convenience samples of residents, family caregivers and professional caregivers. Inclusion criteria were: living in a nursing home (residents), or providing informal or formal care to residents (family caregivers and professional caregivers, respectively). The questionnaires were distributed by e-mail to family caregivers and professional caregivers, and residents were asked to complete the questionnaire on an electronic device (i.e. laptop, notebook or tablet). At least 1325 persons were enrolled: 458 residents, 64 family caregivers, and 803 professional caregivers received the digital questionnaire.

### Analyses

Analysis of the data collected with the QEQ-NH questionnaires was performed with the statistical software of Stata 15 [22]. Subsequently, scale analyses (i.e. factor and reliability analyses, correlations) and regression analyses were used to assess the construct and criterion validity of the three questionnaires. Item-analyses were performed to assess the measurement properties of each item and to select problematic items for cognitive testing.

#### Construct validity: factor structure, scale reliability and inter-scale correlations

Principal Component Analysis with oblimin rotation was conducted. The factor structure of each domain was confirmed with an Eigenvalue of > 1, a Kaiser-Meyer-Oklin value of > 0.60 and when Bartlett’s test of sphericity reached significance (*p* < 0.05). Factor loadings of items should exceed the threshold of >.40. To assess the reliability or internal consistency of the scales found, Cronbach’s alpha was calculated for all factors (domains or scales) of each questionnaire. In classical test theory, a Cronbach’s alpha of 0.7 or higher is indicated as cut-off for a reliable scale [23], but 0.6 is generally recommended as a cut-off value in exploratory analyses [24]. Inter-scale correlations were assessed to determine whether the scales of each questionnaire represent unique and independent constructs, with Pearson correlations being preferably between 0.3 and 0.7 [25].

#### Criterion validity: predictive value of each domain as an indicator for nursing home quality

Criterion validity was investigated by analyzing the relationship between the domains of the QEQ-NH questionnaires (i.e. scale scores) and the Net Promoter Score (NPS) as a global indicator for the perceived quality of nursing home care. Both univariate and multivariate regression analyses were performed with the NPS-question as a continuous (0–10) dependent variable. The analyses indicate whether and to what extent the scores on each domain of the QEQ-NH questionnaire determine the recommendations of nursing homes in each group. Assumptions regarding linearity, collinearity, and homoscedasticity were tested. *P*-values of < 0.05 were regarded as statistically significant.

#### Content validity: item analysis and cognitive testing

The content validity of the questionnaires was tested with item-analyses and cognitive interviews.

##### Item analyses and item selection for cognitive testing

Item- and scale analyses were determined in order to select those questionnaire items that required further testing and optimization with cognitive interviews. These analyses respectively focused on item non-response (% missings), skewness and ceiling or floor effects (extreme distribution of answers), overlap (inter-item correlations), and the contribution of an item to a scale.

The following criteria were used to select problematic items for cognitive testing, based on previous research on questionnaire development and optimization (e.g. [5, 26]):

- Item non-response: > 10% of the answers are either missing or not applicable (indicating that a substantial number of respondents does not understand or could not complete a question);
- Item skewness: > 90% of the answers are in an extreme response category (indicating low variation between cases and little improvement potential in the case of a ceiling effect);
- Item overlap: Pearson correlation between items is > 0.70 (indicating more than 50% overlap in answering patterns and suggesting that one of these items is redundant);
- Item not fitting in or not contributing to a reliable scale: factor loading < 0.40 or Cronbach’s alpha increases if item is deleted from scale (i.e. item does not contribute to a homogeneous set of items for which a reliable composite score can be computed).

##### Cognitive interviews

Cognitive interviews were used to test and refine the problematic questionnaire items, in order to optimize the content validity of the QEQ-NH questionnaires. Cognitive interviewing is a valid and frequently-used technique in the development and testing of questionnaires [26]. It can be used to assess the participants’ comprehension of questions and to identify confusing or unclear wordings, in order to further improve the questionnaires. The interviews were conducted in July 2018, after a period of data collection, and focused on a selection of problematic items of the QEQ-NH questionnaires. Participants were recruited by four nursing homes, representing the three subgroups (i.e. residents, family caregivers, professionals), and they were interviewed in two rounds.

Two researchers (MT and JM) conducted the interviews by using a combination of two interviewing techniques: 1) the think-aloud technique, and 2) the verbal probing technique [27]. Participants were asked to read out loud each question and to describe their thoughts while answering the question. In addition, probes were used to structure the interview and to gain more information on the understanding of the questions. Examples of probes were: ‘*Can you repeat the question in your own words?*’ or ‘*How did you get to this answer?*’. All cognitive interviews were audio-recorded, transcribed verbatim, and content analyzed.

The interviewers (MT and JM) coded problems independently by using the coding system of Willis [27]. This system distinguishes seven categories of questionnaire problems: 1) *clarity* (i.e. problems with the intent or meaning, wording, technical or vague terms), 2) *knowledge* (i.e. trouble remembering or not knowing information, recall or computation problems), 3) *assumptions* (i.e. inappropriate assumptions or underlying logic), 4) *response categories* (i.e. problems with mismatching, missing or overlapping categories), 5) *sensitively* (i.e. sensitive content or wording, socially acceptable), 6) *instructions* (i.e. problems with instructions, introductions or explanations), and 6) *formatting* or lay-out [27]. For each problematic item, the most prominent underlying problem was coded, and sometimes multiple codes were used for one item. All data were entered in a spreadsheet to get an overview of problems per item of each questionnaire; including both qualitative (i.e. verbatim quotations, problem codes, and researchers’ comments) and quantitative data (i.e. number of problems and respondents).

The QEQ-NH questionnaires were optimized, based on the results of the interviews, suggestions of participants and deliberations of the research team. After the first interview round, problem codes were compared and discussed between the two interviewers (MT and JM). Subsequently, questions were adjusted and re-tested in a second interview round, with other participants, to see if the wording or clarifications of these questions indeed had improved. If necessary, items were further optimized after the second interview round. All modifications were discussed and finalized after consensus was reached in the research team (MT, JM and BvdB).

### Informed consent

The nursing homes gave their consent to use the anonymized data for our research purposes. All participants of the cognitive interviews provided a written informed consent, after they received written and verbal information about the cognitive testing. No ethical approval was necessary, as a non-encroaching study like this is not subject to the Dutch Medical Research Involving Human Subjects act (WMO). So, according to Dutch legislation, approval by a medical ethics committee was not mandatory to carry out the research.

## Results

Out of the 1325 persons who were enrolled for the study, a total of 1055 participants completed the questionnaires (response rate 80%), including 359 residents, 48 family caregivers and 648 professional caregivers.

### Construct and criterion validity of the QEQ-NH questionnaires

#### Factor structure and scale reliability

Scale analysis of the QEQ-NH questionnaires revealed four factors with acceptable to good scale reliability for the residents (*Person-centered care*: α = 0.87, *Living and well-being*: α = 0.69, *Safety*: α = 0.70, *Responsive workforce*: α = 0.71), and the same homogenous item sets with slightly different Cronbach’s alphas for the family caregivers (*Person-centered care*: α = 0.85, *Living and well-being*: α = 0.77, *Safety*: α = 0.64, *Responsive workforce*: α = 0.83). Furthermore, factor analysis of the QEQ-NH for professionals revealed seven factors with acceptable to good reliability (Cronbach’s alpha: 0.73 to 0.88). Table 3 shows the number of items and Cronbach’s alpha for each questionnaire scale.

**Table 3:** Number of items and Cronbach’s alpha (α) of the questionnaires’ scales for the three groups

	Residents		Family caregivers		Professional caregivers	
Domains (scales):	No. of items	α	No. of items	α	No. of items	α
1 Person-centered care	8	.87	8	.85	9	.84
2 Living and well-being	7	.69	8	.77	8	.73
3 Safety	3	.70	3	.64	12	.88
4 Learning and improving quality	0	N/A	0	N/A	5	.79
5 Leadership, governance and management	1	N/A	1	N/A	5	.79
6 Responsive workforce	3	.71	3	.83	8	.78
7 Use of resources	0	N/A	0	N/A	5	.75
8 Use of information	1	N/A	1	N/A	2	N/A

*N/A* not applicable (questions did not have to be filled in by this group or Cronbach’s alpha could not be calculated)

#### Inter-scale correlations

Inter-scale correlations for the residents’ and professionals’ questionnaire were good, with Pearson correlations between 0.3 and 0.7 (see Supplementary file 2: Tables 1.1–1.3), confirming independent scales and unique constructs. For the family caregivers’ questionnaire, three scale scores were strongly correlated (*Person-centered care* and *Living and well-being* = 0.76; *Living and well-being* and *Responsive workforce* = 0.78; *p* < 0.01), showing some scale overlap for the domains 1, 2 and 6.

#### Predictive value of each domain as indicator for nursing home global quality rating

In order to test the criterion validity of the QEQ-NH, it was assessed how well the eight scales contribute to the Net Promoter Score (NPS, i.e. recommendations by residents, family and professionals as a global quality indicator of the perceived quality of nursing home care). Almost all assumptions for linear regression were met, except for the homoskedasticity test for the data of family caregivers which showed that the variance of residuals was not equally distributed due to outliers and a small number of respondents. Therefore, the data of family caregivers were not used for the multivariate regression analyses.

Univariate regression analysis, with the NPS-question as a continuous dependent variable (0–10), showed that all scales were significantly and strongly (ß ≥ 0.5) related to recommendation of the nursing home in each group (see Supplementary file 2: Table 2.1).

In the multivariate model, as presented in Table 4, *Person-centered care* (ß = 0.34, *p* < 0.001) and *Living and well-being* (ß = 0.25, *p* = 0.001) remained major significant predictors of the recommendation by residents. Furthermore, *Responsive workforce* (ß = 0.32, *p* < 0.001), *Use of information* (ß = 0.20, *p* < 0.001) and *Leadership, governance and management* (ß = 0.18, *p* < 0.001) were significant predictors of the recommendations by professionals, with *Responsive workforce* being the strongest predictor. The two multivariate regression models explained 53 and 35%, respectively.

**Table 4:** Multivariate regression analysis of QEQ-NH scale scores^#^ as potential predictors of NPS (0–10); unstandardized regression coefficient with 95% confidence interval (B and 95%CI), standardized regression coefficient (ß) and adjusted R^2^ for each multivariate regression model

	Residents (***n*** = 239)		Professional caregivers (***n*** = 579)	
Domains (scales):	B (95% CI)	ß	B (95% CI)	ß
1 Person-centered care	0.77 (0.44–1.10)***	0.34***	− 0,06 (− 0,31 - 0,19)	− 0.03
2 Living and well-being	0,63 (0,27 - 0,98)**	0.25**	−0,04 (− 0,28 - 0,20)	−0.02
3 Safety	0,19 (−0,08 - 0,46)	0.09	−0,10 (− 0,35 - 0,16)	−0.04
4 Learning and improving quality	N/A	N/A	−0,02 (− 0,21 - 0,18)	−0.01
5 Leadership, governance and management	0,17 (0–0,34)	0.11	0,33 (0,17 - 0,50)***	0.18***
6 Responsive workforce	0,07 (−0,14 - 0,29)	0.04	0,67 (0,44 - 0,89)***	0.32***
7 Use of resources	N/A	N/A	0,17 (−0,04 - 0,38)	0.08
8 Use of information	0,07 (−0,07 - 0,21)	0.05	0,34 (0,20 - 0,48)***	0.20***
**Adjusted R**^**2**^		0.53		0.35

^#^ Mean scores per scale, with different number of items and respondents per subgroup

*N/A* not applicable (questions did not have to be filled in by this group)

** Significant relation with NPS (continuous score, 0–10), *p* < 0.01

*** Significant relation with NPS (continuous score, 0–10), *p* < 0.001

### Content validity and questionnaire optimization

#### Item analyses and item selection for cognitive testing

Results of the construct validation analyses and the item non-response analyses were used to select items for further testing with cognitive interviews. Seven out of 23 items (30%) of the residents questionnaire, 11 out of 24 items (46%) of the family questionnaire, and 12 out of the 54 items (22%) for professional caregivers had more than 10% missing values. In addition, six items (in themes 1, 2 and 6) of the questionnaire for family caregivers showed item overlap (Pearson correlation: 0.70 to 0.78). Nevertheless, no item skewness was present in all three questionnaires and the questionnaires for residents and professional caregivers showed no excessive item overlap. Eventually, 11 items (48% of 23 items) of the QEQ-NH for residents, 17 items (71% of 24 items) of the questionnaire for family caregivers and 18 items (33% of 54 items) of the questionnaire for professional caregivers met one or more criteria for further testing, and these items were selected for the cognitive interviews.

#### Participants and interviews in two rounds

A total of 20 participants (7 residents, 5 family caregivers, 8 professional caregivers) were interviewed in two rounds; 11 persons in the first round and nine in the second round. The mean duration of interviews was 26 min (range 11–48 min). The mean age of participants was 84 years (SD = 4.9) for residents, 63 years (SD = 6.3) for family caregivers, and 41 years (SD = 11.8) for professional caregivers. Most participants were female (65%) and the educational level varied from low (25%) to high (35%) (see Supplementary file 2: Table 3.1).

#### Number and type of problems

Table 5 presents the number and type of questionnaire problems in the two interview rounds. The first round (*n* = 11) showed a total of 114 problems for 46 questions (i.e., on average 0.23 problems per question per participant); 18 problems regarding 11 questions in three residents, 31 problems regarding 17 items in three family caregivers, and 65 problems regarding 18 items in five professional caregivers. Considering the various number of participants and questions in each group, the mean number of problems per question per participant was 0.55 for residents, 0.61 for family caregivers, and 0.72 for professionals. Problems in the first interview round mainly concerned clarity (67), and sometimes problems like (a lack of) knowledge (15), assumptions (8), sensitivity (1), or various other problems (23).

**Table 5:** Number and type of questionnaire problems* in the two interview rounds

	**Round 1**		
	Residents (*n* = 3)	Family (*n* = 3)	Professionals (*n* = 5)
**Number of items tested**	11 items	17 items	18 items
**Type of problem**			
Number of clarity problems	14	9	44
Number of knowledge problems	4	8	3
Number of assumptions problems	0	2	6
Number of sensitively problems	0	1	0
Number of other problems	0	11	12
**Total number of problems**	18	31	65
**Mean number of problems per item per respondent**	0.55	0.61	0.72
	**Round 2**		
	Residents (*n* = 4)	Family (*n* = 2)	Professional (*n* = 3)
**Type of problem**			
Number of clarity problems	8	0	10
Number of knowledge problems	4	2	1
Number of assumptions problems	0	0	1
Number of other problems	2	3	1
**Total number of problems**	14	5	13
**Mean number of problems per item per respondent**	0.32	0.15	0.24

* categorized according to the scoring system of Willis (1999) [27]

In the second round (*n* = 9), after refining problematic questions, the total number of problems declined to 32 problems: 14 problems in 4 residents, 5 problems in two family caregivers, and 13 problems in three professionals. The mean number of problems per question per participant in the second round was 0.32 for residents, 0.15 for family caregivers, and 0.24 for professional caregivers respectively. The remaining problems concerned clarity (18), knowledge (7), assumption (1) and some other problems (6). Other problems were for example ambiguous items actually containing two questions, or items with abstract or difficult words that needed more explanation (e.g. by adding examples) or rephrasing (with concrete or simple words).

### Questionnaire optimization

Based on the feedback from the cognitive interviews, more than half of the total number of items of the QEQ-NH were adapted; 15 of the 23 items (65%) for residents, 18 of the 24 items (75%) for family caregivers, and 21 of the 54 items (39%) for the professional caregivers (see Table 6). In addition, one item on *Safety* (domain 3) was deleted and one item on *Leadership, governance and management* (domain 5) was added to both questionnaires for residents and family caregivers. See Supplementary file 2, Box 1.1 for three examples of questions that have been adapted. The revised versions of the QEQ-NH questionnaires (October 2018) consist of 23 items for residents, 24 items for family caregivers, and 54 items for professional caregivers (see Supplementary file 1).

**Table 6:** Results of cognitive interviewing per questionnaire: number of items adapted or deleted

QEQ-NH questionnaire for:	Residents		Family caregivers		Professional caregivers	
Domains:	No. of items adapted	No. of items in final version	No. of items adapted	No. of items in final version	No. of items adapted	No. of items in final version
1 Person-centered care	3	8	5	8	1	9
2 Living and well-being	4	7	4	8	4	8
3 Safety	3^x^	2	3^x^	2	5	12
4 Learning and improving quality	N/A	N/A	N/A	N/A	1	5
5 Leadership, governance and management	2^+^	2	2^+^	2	4	5
6 Responsive workforce	2	3	3	3	2	8
7 Use of resources	N/A	N/A	N/A	N/A	2	5
8 Use of information	1	1	1	1	2	2
Total (% of total items)	15 (65%)	23	18 (75%)	24	21 (39%)	54

^×^ 2 items were adapted and 1 item was deleted

^+^ 1 item was adapted and 1 item was added

## Discussion

This study aimed to validate and optimize the QEQ-NH, a Dutch instrument to measure the quality of care in nursing homes from different perspectives for eight themes of the national quality framework [8]. The QEQ-NH appears to be a solid, comprehensive and promising instrument with an acceptable to good construct, criterion, and content validity. Nonetheless, about half of the items of the three questionnaires needed some adaptations and some items have been deleted after cognitive testing, particularly concerning the questionnaire for family caregivers. This optimization is likely to have yielded an even better, valid and reliable instrument, but future research should reveal whether the questionnaires are adequately optimized for measuring, improving and monitoring the quality of nursing home care.

### Acceptable to good psychometric properties

Testing the three questionnaires showed robust scales and independent or unique constructs, with Cronbach’s alphas varying between .69 and .88 (except for *Safety* in the questionnaire for family caregivers, which is no longer a scale as an item has been deleted), and Pearson inter-scale correlations between .30 and .70 (except for three domains in the questionnaire for family caregivers showing some overlap, with correlations of .76 and .78). Furthermore, each scale of the questionnaires was significantly related to the generic quality measure, i.e. the recommendation question (Net Promoter Score), proving criterion validity. With respect to the content validity, however, about half of the items (11 to 18 items per questionnaire) were further tested, because of excessive missing values or redundancy due to item overlap. In the cognitive interviews, these problematic items particularly showed clarity and knowledge problems, and consequently have been adapted.

The psychometric analysis of the QEQ-NH showed good internal consistency or reliability for almost all scales (Cronbach’s alpha > 0.7). The internal consistency of two scales was somewhat lower, i.e. *safety* in the QEQ-NH for family caregivers (α = 0.64) and *living and well-being* in the questionnaire for residents (α = 0.69), but the Cronbach’s alphas were still acceptable.

The psychometric properties of the questionnaire for family caregivers were the least optimal and this questionnaire needed most adaptations because of item-overlap, many missing values and various problems shown by the cognitive interviews. Family caregivers reported the most ‘knowledge problems’ during the cognitive interviews, compared to the other two groups, which relate to difficulties in answering a question due to a lack of information [27]. A possible explanation for this could be that family caregivers are less involved in the daily care for their relatives, and that they might be relatively unaware of the specific care process and activities in the nursing home. Consequently, family caregivers are likely to have most difficulty in rating the quality of nursing home care and it is hard to capture the experiences or perspective of family caregivers well. Nevertheless, the cognitive interviews offered many clues for item optimization and seemed to have resulted in a more valid and feasible questionnaire since the second interview round showed far less problems.

### Monitoring and improving quality from different perspectives

In the past two decades, many instruments have been developed to assess quality of care in nursing homes. However, most instruments either focus on specific quality domains or on specific groups of residents. The QEQ-NH is a relatively new instrument for evaluating the quality of care comprehensively and multi-dimensionally, from different perspectives. It enables the monitoring and evaluation of the various domains of quality of care in nursing homes, for all themes of the national quality framework in the Netherlands, and for all types of residents. It can be applied in an everyday care setting in order to continuously monitor and improve the care in nursing homes, based on real-time feedback. The instrument is increasingly being used for nationwide quality evaluations or ‘quality scans’ in nursing homes. National data in particular show potential areas of quality improvement concerning the preconditions ‘Staff composition’ and ‘Leadership’, as well as for the themes ‘Safety’ and ‘Learning and improving’ in nursing home care [28].

The results of our study confirm the capacity of the instrument to show potential areas for quality improvement. Nonetheless, residents, family caregivers and professional caregivers might differ in their evaluations and opinions regarding ‘good quality of care’. Therefore, it is important to be aware of different perspectives or preferences and to take into account the different views and opinions in the management and improvement of care. It is also important to carefully interpret the questionnaires’ outcomes, as participants might overrate or underrate the quality of care. For example, residents might overrate the quality of care due to dependence or factors such as not feeling safe nor being able to speak freely [29]. Additional conversation and in-depth interviews with different stakeholders could help to determine how to further improve the quality of care.

### Various stakeholders rate quality differently

The results of our study show that residents, family caregivers and professional caregivers are generally likely to differ in their evaluation of the quality of care. Interestingly, a varying impact of the eight themes was found in predicting the overall perceived quality of care for the three perspectives as measured by the NPS recommendation-question. Residents rated *person-centered care* as the most important theme for a good quality of care, whereas a *responsive workforce,* the *use of information* and *leadership* were the most important predictors of quality of care according to professionals. These findings do not only imply that various stakeholders might perceive or interpret quality differently, but also suggest that the experiences or assumptions underlying the overall perceived quality actually differ between subgroups. In other words, the concept quality of care is likely to be defined differently by various stakeholders. Future studies with the QEQ-NH could reveal whether the perceived quality of care in nursing homes and the key areas for quality improvement actually differ between subgroups.

Literature confirms the different views of various stakeholders on the quality of nursing home care. For example, Kahdka et al. (2020) found that proxies generally perceived lower quality of care experience in comparison with older people themselves [16]. Furthermore, results of a Dutch nationwide improvement program for the elderly showed that older adults evaluated the quality of care less positively than professionals, particularly in evaluating the person-centeredness of care [30]. We also found the QEQ-NH theme *person-centered care* to be the most important predictor of the perceived quality of care in residents. In contrast, professionals rated preconditions such as having a *responsive workforce* and *leadership, governance and management* as the most important predictors of quality of care. Other research shows that factors related to the work environment, such as team climate and collaboration between team members, contribute to the quality of care in nursing homes [31, 32]. Thus, in order to improve care from a residential and professional perspective, it seems crucial to focus both on person-centered care and on preconditions of care, as these factors are strongly related to the overall quality of care in nursing homes.

For family caregivers, the QEQ-NH theme *safety* was most important in predicting good quality of care. This finding is in line with the policy of the health inspectorate in the Netherlands to warrant safety as a basic prerequisite for good quality of care in nursing homes. Previous research also showed that family caregivers particularly emphasize the need to improve the safety of the living environment [5]. However, assessments by family caregivers should be interpreted with caution as their judgement may be influenced by factors that are not related to the caregiving situation, i.e., a general opinion on nursing homes or emotional feelings such as guilt or sadness because of their loved ones living in nursing homes [33, 34]. Therefore, the perspective of family caregiver on the quality of care in nursing homes must be interpreted carefully and is a subject for further study.

### Study limitations

The present study has some limitations. Firstly, the participating nursing homes took part in a quality improvement program in the Netherlands because they had distinct quality issues. Secondly, the participants who filled in the questionnaire were not randomly selected and a selection bias might have been introduced by professionals who invited residents to participate and who distributed the questionnaires among family caregivers. Thirdly, the number of family caregivers who completed a questionnaire was relatively low, so the psychometric properties of this version of the QEQ-NH should be reassessed in future research. Another study limitation concerns the use of the recommendation question of the Net Promotor Score because this global indicator might not be the best criterion or gold standard for the quality of nursing home care. The NPS reflects how likely respondents are to promote the nursing home but seems to be less valid than a global rating [21]. In addition, since the background and response characteristics of participants were not registered, and because questionnaires were not personalized and completed anonymously, we were not able to describe the response and demographic features of participants, nor able to verify the representativeness of the data. Thus, the study setting is not likely to be representative for all nursing homes in the Netherlands and the respondents might have under- or overrated the quality of care in the nursing homes. However, irrespective of the level of quality in the participating nursing homes, this study validated and optimized an instrument that can be used to evaluate and improve quality in all nursing homes.

### Future research

The Quality Evaluation Questionnaires are completed annually in a wide variety of nursing homes in the Netherlands, so more data will be collected that can be used for answering remaining research questions. We expect the optimized instrument to show even more relevant, valid and reliable quality scores in future measurements. Nonetheless, future research is necessary to determine whether the psychometric properties of the optimized versions of the QEQ-NH questionnaires have actually been improved. These future studies should ensure more representative study settings and participants, with acceptable sample sizes for all subgroups, and could also confirm the wider usability and feasibility of the instrument.

Additional research with repeated measurements is needed to show whether this instrument could actually contribute to the quality of care by identifying focus points for improvement efforts, and if it can be used to monitor changes over time. The responsiveness of questionnaires should be evaluated, for instance by relating change scores to actual improvement efforts that have been implemented in between two measurements. Furthermore, data is needed to study the reliability and discriminatory power of the questionnaires, to get insight into possible (selection) bias and confounders (e.g. the frequency of visits by family caregivers influencing their perceived quality), and to explore whether the instrument can be used for comparisons between teams, across facilities or in nationwide benchmark studies. Finally, future research could help to reveal what is actually needed to improve the quality of care in nursing homes, and to further investigate the differences in views and ratings of stakeholders in order to improve the quality of care from various perspectives.

## Conclusions

In conclusion, the Quality Evaluation Questionnaires for Nursing Homes (QEQ-NH) appears to be a promising and valid instrument for measuring the quality of nursing home care. This comprehensive instrument is designed for evaluating, improving and monitoring the quality of care in nursing homes from various perspectives, by covering the national quality themes and the views of all stakeholders involved. This study shows that the QEQ-NH has overall good psychometric quality, with an acceptable to good construct, criterion and content validity. Nonetheless, cognitive testing showed several problems in answering the questionnaires and future research should prove whether the optimization resulted in even more valid and reliable questionnaires. Results also show that residents, family caregivers and professional caregivers are likely to differ in their values and evaluations of the quality of care in nursing homes. Future research should demonstrate the improved psychometric properties of the optimized questionnaires and the wider usability and feasibility of the QEQ-NH for improving and monitoring the quality of care in nursing homes.

## Supplementary Information

**Additional file 1.**

**Additional file 2.**

## Data Availability

The datasets used and/or analyzed during the current study are available from the corresponding author on reasonable request.

## Abbreviation

QEQ-NH: Quality evaluation questionnaires for nursing homes
