# Supplementary material for: Quality evaluation questionnaires – nursing homes (QEQ-NH); validation of questionnaires for measuring quality of care in nursing homes from various perspectives
Source: BMC Health Serv Res. 2021 Sep 13;21:961. doi: 10.1186/s12913-021-06823-4 (PMC8436484; doi:10.1186/s12913-021-06823-4)
Supplement: Supplementary file 1 — Additional file 1. [file 12913_2021_6823_MOESM1_ESM.docx]

**SUPPLEMENTARY FILE 1**

**Domains and items of three QEQ-NH questionnaires**

Final versions of the questionnaires include: 23 items for residents, 24 items for family caregivers, and 54 items for professional caregivers. Questions are formulated as propositions and answering scales range from 1 (completely disagree) to 5 (completely agree).

| **Domains:** | **Residents** | **Family caregivers** | **Professional caregivers** |
| --- | --- | --- | --- |
| **1. Person-centered care** |  |  |  |
| 1.1    Attention for each client and their unique situation | x | x | x |
| 1.2    Background of client is known and respected | x | x | x |
| 1.3    Needs and preferences are listened to | x | x | x |
| 1.4    Shared decisions and agreements are made about daily care | x | x | x |
| 1.5    Clients can influence the type and timing of care as well as daytime activities | x | x | x |
| 1.6    Ongoing comprehensive assessment of clients’ needs is taken into account |  |  | x |
| 1.7   Personal care plan is set up together with client | x | x | x |
| 1.8    Personal care plan reflects the clients’ needs and preferences | x | x | x |
| 1.9    Evaluation of personal care plan | x | x | x |
| **2. Living and well-being** |  |  |  |
| 2.1    Support for personal healthcare needs and attention for personal background | x | x | x |
| 2.2    Recreational and stimulating activities | x | x | x |
| 2.3    Care for physical well-being | x | x | x |
| 2.4    Participation of family caregivers |  | x | x |
| 2.5    Working together with volunteers | x | x | x |
| 2.6    Clean and safe housing | x | x | x |
| 2.7    Appropriate and nice meals | x | x | x |
| 2.8    Clients’ privacy is respected | x | x | x |
| **3. Safety** |  |  |  |
| 3.1   Professionals adhere to the medication guidelines |  |  | x |
| 3.2    Prevention and monitoring health risks of skin |  |  | x |
| 3.3   Professionals adhere to the service’s and national policy of restrictions of freedom restraint |  |  | x |
| 3.4    Prevention from acute risk of harm and hospitalization |  |  | x |
| 3.5    Professionals work according to the residential services policies |  |  | x |
| 3.6    Prevention and control practices of health risks |  |  | x |
| 3.7   Monitoring and evaluation of health risks |  |  | x |
| 3.8    Prevention of incidents (e.g. medication errors) |  |  | x |
| 3.9    Reporting and investigating notifiable incidents adverse events |  |  | x |
| 3.10 Professionals work according to an excellent hygienic standard | x | x | x |
| 3.11 High risk work is done by qualified health care professionals |  |  | x |
| 3.12 Coordination of care between different professionals and disciplines | x | x | x |
| **4. Learning and improving quality** |  |  |  |
| 4.1    Continuously attention for improvement of quality of care |  |  | x |
| 4.2    Giving feedback to co-workers |  |  | x |
| 4.3    Evaluation of and learning from incidents and adverse events |  |  | x |
| 4.4    Use of (quality) information to improve care |  |  | x |
| 4.5    Possibility for consultation |  |  | x |
| **5. Leadership, governance and management** |  |  |  |
| 5.1 Statement of purpose for the residential service | x | x | x |
| 5.2 The statement of purpose is incorporated in the daily activities |  |  | x |
| 5.3 Leadership is supporting, facilitating and transparent |  |  | x |
| 5.4 Clients and their family members are involved in the services’ policy and regulations | x | x | x |
| 5.5 Professionals are involved in relevant standards and regulations |  |  | x |
| **6. Responsive workforce** |  |  |  |
| 6.1    Sufficient numbers and quality of health care professionals |  |  | x |
| 6.2    Training and ongoing development opportunities for staff |  |  | x |
| 6.3    Sufficient time to deliver person-centered care | x | x | x |
| 6.4    Balance between permanent and temporary staff | x | x | x |
| 6.5    Sufficient specialists in team |  |  | x |
| 6.6    Good coordination of tasks, responsibilities and authorizations |  |  | x |
| 6.7    Teams work well together | x | x | x |
| 6.8    Person in charge is available for supervision of all team members |  |  | x |
| **7. Use of resources** |  |  |  |
| 7.1    Procedures that support the daily work |  |  | x |
| 7.2    Continuous evaluation of procedures to improve care |  |  | x |
| 7.3    (Protective) materials and tools are available |  |  | x |
| 7.4    Use of technological tools and applications |  |  | x |
| 7.5    Facility services support process of care |  |  | x |
| **8. Use of information** |  |  |  |
| 8.1    System to gather information about client experiences regarding quality of care and safety | x | x | x |
| 8.2    Information about quality and safety of the residential service is shared to drive for continuous improvements |  |  | x |
| **Total number of items** | **23** | **24** | **54** |
