# Supplementary material for: Quality evaluation questionnaires – nursing homes (QEQ-NH); validation of questionnaires for measuring quality of care in nursing homes from various perspectives
Source: BMC Health Serv Res. 2021 Sep 13;21:961. doi: 10.1186/s12913-021-06823-4 (PMC8436484; doi:10.1186/s12913-021-06823-4)
Supplement: Supplementary file 2 — Additional file 2. [file 12913_2021_6823_MOESM2_ESM.docx]

**SUPPLEMENTARY FILE 2**

**Additional tables 1.1 to 3.1 and box 1.1.**

**Table 1.1** Residents questionnaire: inter-scale correlations

| **Scales (domains):** | 1 | 2 | 3 | 5 | 6 | 8 |
| --- | --- | --- | --- | --- | --- | --- |
| 1 Person-centered care | 1.00 |  |  |  |  |  |
| 2 Living and well-being | 0.68*** | 1.00 |  |  |  |  |
| 3 Safety | 0.61*** | 0.63*** | 1.00 |  |  |  |
| 5 Leadership, governance and management | 0.52*** | 0.48*** | 0.42*** | 1.00 |  |  |
| 6 Responsive workforce | 0.66*** | 0.63*** | 0.52*** | 0.46*** | 1.00 |  |
| 8 Use of information | 0.44*** | 0.36*** | 0.32*** | 0.38*** | 0.34*** | 1.00 |

***p<0.001

**Table 1.2** Family caregivers questionnaire: inter-scale correlations (r>0.70 are printed in bold)

| **Scales (domains):** | 1 | 2 | 3 | 5 | 6 | 8 |
| --- | --- | --- | --- | --- | --- | --- |
| 1 Person-centered care | 1.00 |  |  |  |  |  |
| 2 Living and well-being | **0.76***** | 1.00 |  |  |  |  |
| 3 Safety | 0.69*** | 0.63*** | 1.00 |  |  |  |
| 5 Leadership, governance and management | 0.34* | 0.50*** | 0.26 | 1.00 |  |  |
| 6 Responsive workforce | 0.59*** | **0.78***** | 0.61*** | 0.44** | 1.00 |  |
| 8 Use of information | 0.69*** | 0.61*** | 0.41* | 0.44** | 0.52** | 1.00 |

**p<0.01

***p<0.001

**Table 1.3** Professional caregivers questionnaire: inter-scale correlations

| **Scales (domains):** | 1 | 2 | 3 | 4 | 5 | 6 | 7 | 8 |
| --- | --- | --- | --- | --- | --- | --- | --- | --- |
| 1 Person-centered care | 1.00 |  |  |  |  |  |  |  |
| 2 Living and well-being | 0.66*** | 1.00 |  |  |  |  |  |  |
| 3 Safety | 0.68*** | 0.57*** | 1.00 |  |  |  |  |  |
| 4 Learning and improving quality | 0.63*** | 0.63*** | 0.64*** | 1.00 |  |  |  |  |
| 5 Leadership, governance and management | 0.49*** | 0.47*** | 0.46*** | 0.57*** | 1.00 |  |  |  |
| 6 Responsive workforce | 0.62*** | 0.61*** | 0.56*** | 0.68*** | 0.60*** | 1.00 |  |  |
| 7 Use of resources | 0.58*** | 0.59*** | 0.55*** | 0.64*** | 0.58*** | 0.66*** | 1.00 |  |
| 8 Use of information | 0.44*** | 0.40*** | 0.41*** | 0.43*** | 0.51*** | 0.49*** | 0.54*** | 1.00 |

***p<0.001

**Table 2.1** Univariate regression analysis of QEQ-NH scale scores^#^ as predictors of the NPS (0-10): standardized regression coefficient (ß) of each domain as a predictor of the recommendation score

|  | **Residents** | | **Family**  **caregivers** | | **Professional caregivers** | |
| --- | --- | --- | --- | --- | --- | --- |
| **Domains (scales):** | **n** | **ß** | **n** | **ß** | **n** | **ß** |
| 1 Person-centered care | 354 | **1.39***** | 48 | **1.10**** | 641 | **0.88***** |
| 2 Living and well-being | 355 | **1.51***** | 48 | **1.12**** | 642 | **0.93***** |
| 3 Safety | 349 | **1.04***** | 46 | **1.40***** | 641 | **0.84***** |
| 4 Learning and improving quality | 0 | N/A | 0 | N/A | 642 | **0.76***** |
| 5 Leadership, governance and management | 308 | **0.79***** | 44 | **0.60*** | 640 | **0.88***** |
| 6 Responsive workforce | 350 | **0.85***** | 45 | **0.86**** | 644 | **1.13***** |
| 7 Use of resources | 0 | N/A | 0 | N/A | 643 | **0.97***** |
| 8 Use of information | 275 | **0.47***** | 38 | **0.56*** | 586 | **0.78***** |

^#^ Mean scores per scale and the number of items and respondents differ across subgroups; N/A=not applicable (questions did not have to be filled in by this group)

* Significant relation with NPS (continuous score, 0-10), p<0.05

** Significant relation with NPS (continuous score, 0-10), p<0.01

*** Significant relation with NPS (continuous score, 0-10), p<0.001

**Table 3.1** Characteristics of participants in 20 cognitive interviews (total of two interview rounds)

|  | **Residents** | | | **Family caregivers** | | | **Professional caregivers** | | |
| --- | --- | --- | --- | --- | --- | --- | --- | --- | --- |
|  | **Round 1 (n=3)** | **Round 2 (n=4)** | **Total**  **(n=7)** | **Round 1 (n=3)** | **Round 2 (n=2)** | **Total (n=5)** | **Round 1 (n=5)** | **Round 2 (n=3)** | **Total (n=8)** |
| Mean age (SD) | 85 (4.9) | 82 (10) | 84 (8.4) | 63 (5.7) | 63 (7) | 63 (6.3) | 38 (12) | 45 (9.9) | 41 (11.8) |
| Gender: |  |  |  |  |  |  |  |  |  |
| Female (%) | 2 (67) | 3 (75) | 5 (71) | 0 (0) | 1 (50) | 1 (20) | 5 (100) | 2 (67) | 7 (88) |
| Education level* (%): |  |  |  |  |  |  |  |  |  |
| Low | 2 (67) | 2 (50) | 4 (57) | 0 (0) | 0 (0) | 0 (0) | 0 (0) | 1 (33) | 1 (13) |
| Medium | 1 (33) | 1 (25) | 2 (29) | 0 (0) | 2 (100) | 2 (40) | 2 (40) | 2 (67) | 4 (50) |
| High | 0 (0) | 1 (25) | 1 (14) | 3 (100) | 0 (0) | 3 (60) | 3 (60) | 0 (0) | 3 (38) |

* Low (4 years secondary education), Medium (5 years secondary education), or High (≥6 years secondary education)

**Box 1.1** Examples of questionnaire problems and solutions

**Example 1: Clarity problem**

Original question (in questionnaire for Professional caregivers):

**My team discusses and uses relevant quality information in order to learn from it.**

Almost all of the interviewed professionals struggled with the meaning of ‘quality information’ (“*hm … relevant quality information … what is relevant quality information?*”). In addition, the word ‘relevant’ was unclear to participants , i.e., why is some quality information relevant? We modified the question, by deleting the word ‘relevant’ and by clarifying the meaning of ‘quality information’, into: **My team discusses and uses quality information and signals to learn from. Quality information includes for example: internal audits, reports on incidents or complaints, and client satisfaction measures.**

**Example 2: Knowledge problem**

Original question (in questionnaire for Residents and Family caregivers):
**Professional caregivers always report incidents and almost-incidents.**

All participants experienced difficulties in answering this question, because they missed information. Residents and family caregivers don’t know whether professionals do report incidents or not. That’s why this question was deleted from the questionnaire for residents and family caregivers.

**Example 3: Assumption problem**

Original question (in questionnaire for Professionals):

**My team works together with volunteers. It is clear to volunteers and other parties involved what is allowed or not allowed.**

This question suggests that every nursing home does work with volunteers, whereas participants stressed that this is not the case and that nursing homes are not obliged to work with volunteers. In addition, ‘other parties’ was not clear. The question was modified into: **Volunteers and family members are available to help the professional caregivers in conducting activities.**
